# The Effect of Coupling Agents and Graphene on the Mechanical Properties of Film-Based Post-Consumer Recycled Plastic

**DOI:** 10.3390/polym16030380

**Published:** 2024-01-30

**Authors:** Sungwoong Choi, Jianxiang Zhao, Patrick C. Lee, Duyoung Choi

**Affiliations:** 1 Carbon & Light Materials Group, Korea Institute of Industrial Technology, Jeonju 54853, Republic of Korea; 2 Division of Mechanical Design Engineering, Jeonbuk National University, Jeonju 54896, Republic of Korea; 3 Multifunctional Composites Manufacturing Laboratory (MCML), Department of Mechanical and Industrial Engineering, University of Toronto, Toronto, ON M5S 3G8, Canadapatricklee@mie.utoronto.ca (P.C.L.)

**Keywords:** recycling, recycled plastic, polymer composites, coupling agent, maleic anhydride, nanofiller, graphene, post-consumer recycled plastic (PCR)

## Abstract

This study aims to improve the mechanical properties of post-consumer recycled (PCR) plastic composed primarily of polypropylene (PP) and polyethylene (PE), which generally exhibit poor miscibility, by applying coupling agents and graphene. Here, we compare a commercially available coupling agent with a directly synthesized maleic anhydride (MA) coupling agent. When applied to a 5:5 blend of recycled PP and PE, an optimum tensile strength was achieved at a 3 wt% coupling agent concentration, with the MA coupling agent outperforming the commercial one. Characterization through Fourier transform infrared spectroscopy (FT-IR) and thermogravimetry analysis (TGA) revealed a PP:PE ratio of approximately 3:7 in the PCR plastics, with 4.86% heterogeneous materials present. Applying 3 wt% of the commercial and MA coupling agents to the PCR plastics resulted in a significant 53.9% increase in the tensile strength, reaching 11.25 MPa, and a remarkable 421.54% increase in the melt flow index (MFI), reaching 25.66 g/10 min. Furthermore, incorporating 5 wt% graphene led to a notable 64.84% increase in the tensile strength. In addition, the application of MA coupling agents and graphene improved the thermal stability of the PCR plastics. These findings show significant promise for addressing environmental concerns associated with plastic waste by facilitating the recycling of PCR plastics into new products. The utilization of coupling agents and graphene offers a viable approach to enhance the mechanical properties of PCR plastics, paving the way for sustainable and environmentally friendly solutions.

## 1. Introduction

The development of the plastic industry has had a significant effect on human life and the growth of high-tech industries such as automobiles [1], space [2], and aviation [3]. The rapid increase in plastic usage has inevitably led to an increase in plastic waste [4,5,6]. Plastic waste recycling in the EU only accounts for about 5–10% of the total plastic demand. Overall, post-consumer recycled (PCR) plastic waste accounts for 49% of plastic production, of which only 32.5% is recycled. The remaining 25% is landfilled, and 42.5% is recovered for energy. Much plastic waste is still exported to developing countries, some hidden in untracked trade flows or illegal landfills [7]. As a result, this plastic waste is typically landfilled or incinerated, causing harm to the natural environment [8,9]. Film packaging, primarily used for food, is among the primary contributors to plastic waste. High-density polyethylene (HDPE), polypropylene (PP), polyvinyl chloride (PVC), and polystyrene (PS) are commonly utilized for film packaging [10,11]. Certain materials, like PP, exhibit high resistance to photodegradation and may take up to 1000 years to degrade. Non-degradable plastics are one of the leading causes of environmental pollution. Consequently, there is a global interest in addressing this issue through various approaches, such as plastic waste reduction, recycling, and pyrolysis [12,13].

In addition to the commonly used methods of landfill and incineration, there are also methods of recycling plastic waste through simple recycling and mixing [14]. In general, plastic waste consists of a mixture of PP, polyethylene (PE), polyethylene terephthalate (PET), and other materials that are incompatible with each other due to differences in their polarity, resulting in the formation of a multisystem. Therefore, it is challenging to achieve good physical properties through simple mixing [15]. Therefore, during the recycling process, the plastic loses its mechanical properties and melt flow index (MFI). In industrial applications, the MFI is important to consider due to the high production rates. In terms of process efficiency, MFI improvement is related to flowability, production rate, and miscibility [16,17,18]. The mechanical properties and MFI of plastics waste can be improved by using coupling agents [19,20]. Ahmadlouydarab et al. investigated the effect of polypropylene–graft–maleic anhydride (PP-g-MA) on the properties of recycled polyethylene terephthalate (rPET)/PP blends. As a result, the PP-g-MA exhibited improved compatibility and mechanical properties [21]. Tucker et al. investigated the effects of PP-g-MA and styrene–ethylene–butylene–styrene-grafted MA (SEBS-g-MA) on PP/nylon 6 blends. As a result, both coupling agents showed a positive effect on improving the mechanical properties of the PP/nylon 6 blends [22].

Another way to enhance the mechanical properties is by incorporating nanofillers into a polymer matrix [23]. Graphene, a remarkable two-dimensional nanomaterial with a flat structure composed of carbon atoms, is incredibly desirable due to its outstanding strength, high conductivity, and flexibility. The exceptional intrinsic bonding of graphene can be utilized to improve mechanical and thermal properties in various fields. Moreover, the thin structure of graphene makes it suitable for diverse applications. Introducing graphene can not only improve product quality but also enhance the efficiency of recycling processes and improve thermal properties, which increases process efficiency and further improves quality. This will ultimately reduce environmental burdens and contribute to the development of sustainable recycling systems. In this regard, research has been conducted to improve mechanical properties using graphene. Kar et al. improved mechanical properties by adding poly(methyl methacrylate) (PMMA), which adds compatibility with graphene oxide (GO), to a mixture of poly(vinylidene fluoride) (PVDF) and acrylonitrile butadiene styrene (ABS) [24]. Cao et al. applied graphene oxide sheets (GOSs) to immiscible polymers such as polyamide (PA) and polyphenylene oxide (PPO). The GOSs not only improved compatibility but also improved mechanical and thermal properties [25].

The objective of this study is to improve the mechanical properties and MFIs of PCR plastic based on film packaging materials by applying coupling agents and graphene. We produced maleic anhydride (MA) coupling agents in our lab Firstly, the optimization of the coupling agents and graphene was conducted using recycled polypropylene (rPP) and recycled polyethylene (rPE). Then, the optimized compositions were applied to the PCR plastic. The characteristics of the PCR plastic were investigated through Fourier transform infrared spectroscopy (FT-IR) and thermogravimetry analysis (TGA), while the effects of the coupling agents and graphene were examined by analyzing the tensile strength and MFI. The results of this study can contribute to addressing environmental issues by applying the improved mechanical strength and MFI of PCR plastics in various industries.

## 2. Materials and Methods

### 2.1. Materials

**Figure 1:**
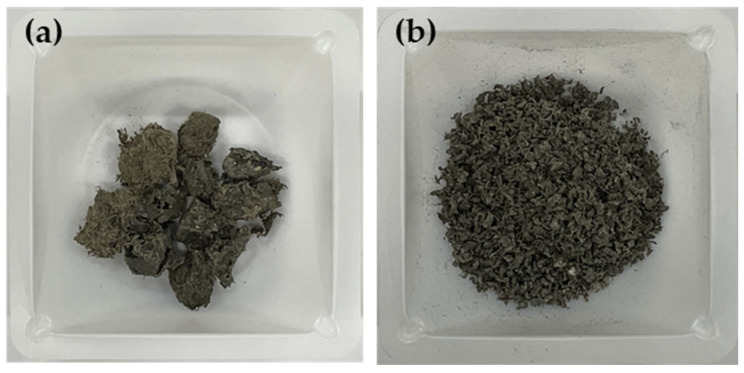
Freeze crushing PCR plastic. (**a**) PCR plastic raw materials and (**b**) PCR plastic material after 2 mm mesh freeze crushing.

**Figure 2:**
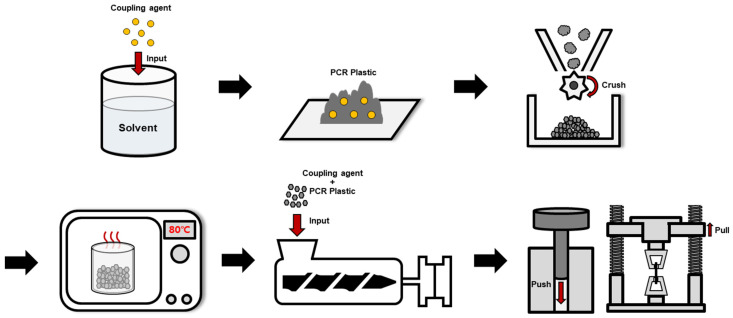
Schematic overview of the preparation process for PCR plastic specimens with coupling agent application.

The rPP and rPE were purchased from Sarom ENG (Hwaseong, Republic of Korea). The film-based PCR plastic used in this study was provided by Cheongsol CNT Co., Ltd., (Goyang, Republic of Korea). The PCR plastic was crushed into 2 mm particles using a freezer mill (Universal cutting mill, Taemyung Science, Uiwang, Republic of Korea) before being used. The raw PCR plastic and the 2 mm crushed PCR plastic are depicted in Figure 1. Table 1 shows the characteristics and sources of the commercial coupling agents applied to rPP and rPE, and Table 1 shows the characteristics and sources of the commercial coupling agents applied to rPP, rPE, and the PCR plastic. MA and dicumyl peroxide (DCP) were purchased from Sigma Aldrich (St. Louis, MO, USA) in the USA. Graphene with an average particle size of 100–300 μm was obtained from New Graphene World (Hwaseong, Republic of Korea). Figure 2 shows an overview of the preparation process for the PCR plastic specimens with the application of coupling agents. Table 2 shows the content of MA, DCP, and graphene applied to the PCR plastic. First, the commercial coupling agents and graphene were directly applied to the PCR plastic without any treatment. MA and DCP were dissolved in a solvent at the concentrations indicated in Table 2 to prepare the MA coupling agent. After evaporating the solvent, the MA coupling agent was mixed with rPP, rPE, and the PCR plastic. The mixture was then crushed into 2 mm particles using a cryogenic grinder. To remove moisture from the crushed specimens, they were dried in a vacuum oven set at 80 °C for 2 h. The dried specimens were then molded into tensile specimens using a hot press set at 230 °C and a pressure of 500 bar.

### 2.2. Methods

#### 2.2.1. Thermogravimetry Analysis (TGA)

The content of heterogeneous substances and thermal properties of the PCR plastic were analyzed using TGA with a TA Instruments SDT 650 (New Castle, DE, USA) instrument. The sample weight was set to approximately 5–10 mg, and each sample was heated at a rate of 10 °C/min in a nitrogen atmosphere up to 650 °C. After the measurement, the remaining weight and content of the heterogeneous substances in the PCR plastic, as well as the thermal degradation temperature, were investigated.

#### 2.2.2. Fourier Transform Infrared Spectroscopy (FT-IR)

FT-IR spectra were measured using Thermo Fisher Scientific’s FT-IR Nicolet iS50 (Waltham, MA, USA) spectrometer in reflectance mode. The spectra were acquired using the Attenuated Total Reflection (ATR) technique, which measures the reflectance infrared spectra. Spectra were recorded in the wavenumber range of 550–4000 cm^−1^ with a spectral resolution of 8 cm^−1^ and averaged over 16 scans. A quantitative analysis of the area under the measured FT-IR spectra was performed to determine the content of PP and PE in the PCR plastic as well as the grafting ratio of the coupling agent.

#### 2.2.3. Melt Flow Index (MFI)

The MFI analysis was conducted using the Dynisco LMI5000 instrument (Franklin, MA, USA). The measurements were performed under the conditions of 230 °C and a load of 2.16 kg in the ASTM D1238 [26]. At least five specimens were measured for each sample, and the average value was determined. Prior to the MFI measurements, the specimens were dried in a vacuum oven at 80 °C for 2 h to remove residual moisture.

#### 2.2.4. Tensile Strength

Tensile testing was conducted using a universal testing machine (Instron 3382, Instron, Norwood, MA, USA). The measurements were performed at room temperature (25 °C). At least five specimens were tested for each sample, and the average value was calculated. The gauge length of the specimens was set at 50 mm, and the crosshead speed was set at 5 mm/min.

## 3. Results and Discussion

### 3.1. Characterization of PCR Plastics

**Figure 3:**
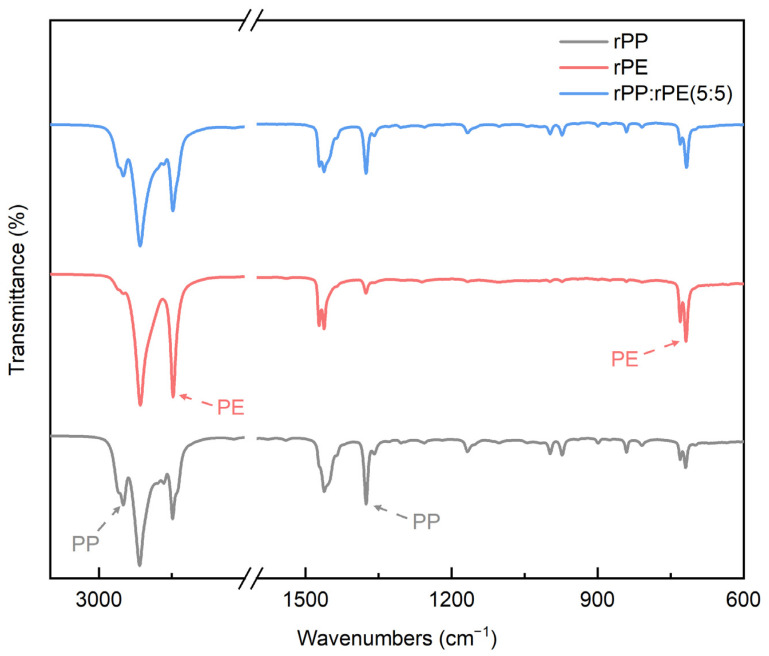
FT-IR spectra of rPP, rPE and rPP:rPE (5:5) samples.

Figure 3 shows the peaks of the reference rPP, rPE, and the FT-IR spectra of the rPP:rPE (5:5) sample. The obtained peaks show the typical spectra of PP and PE [27]. The specific PP peaks are shown at 2950 and 1375 cm^−1^, and the specific PE peaks are shown at 2847 and 718 cm^−1^. The peaks at 2950 and 2847 cm^−1^ are intensive absorption bands due to the symmetrical and asymmetric stretching vibrations of the C–H bonds such as CH_2_ and CH_3_. The peak at 1375 cm^−1^ is assigned to the CH_2_ and CH_3_ transformation oscillations of aliphatic groups in the polymer chain. The peak at 718 cm^−1^ is associated with the rocking vibrations of PE macromolecules [28,29,30,31,32]. Compared to the reference peak, at the rPP:rPE (5:5) sample, the characteristic peaks of rPP and rPE are shown. In particular, the 2950 cm^−1^ peak of the rPP:rPE (5:5) sample shows that PE is affected by PP. The values of the areas of the said peaks compared by a quantitative analysis are shown in Table 3, and the content of the rPP:rPE (5:5) sample was calculated by comparing these areas. The content was approximately 48.75% PP and 51.25% PE. Considering errors in the dispersion process, the ratio of rPP to rPE was close to 5:5.

**Figure 4:**
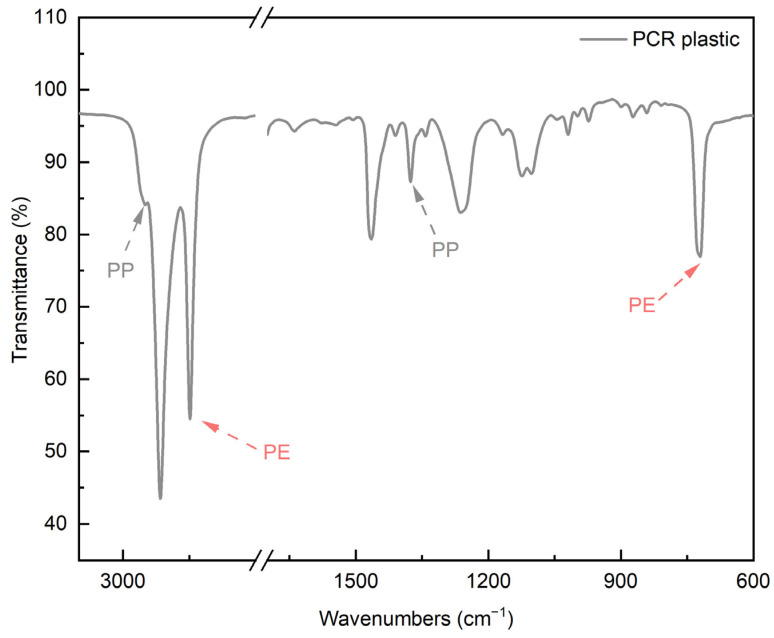
FT-IR spectra of PCR plastic.

**Figure 5:**
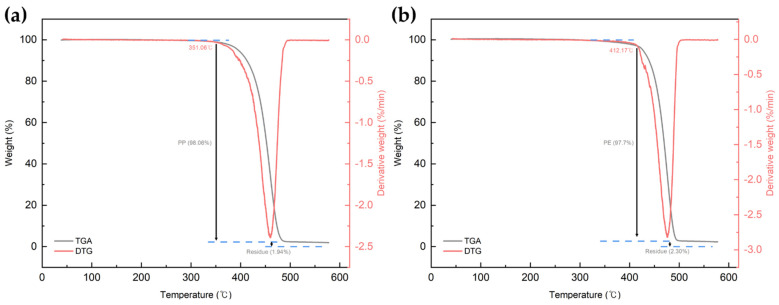
TGA and DTG measurement results. (**a**) rPP and (**b**) rPE.

**Figure 6:**
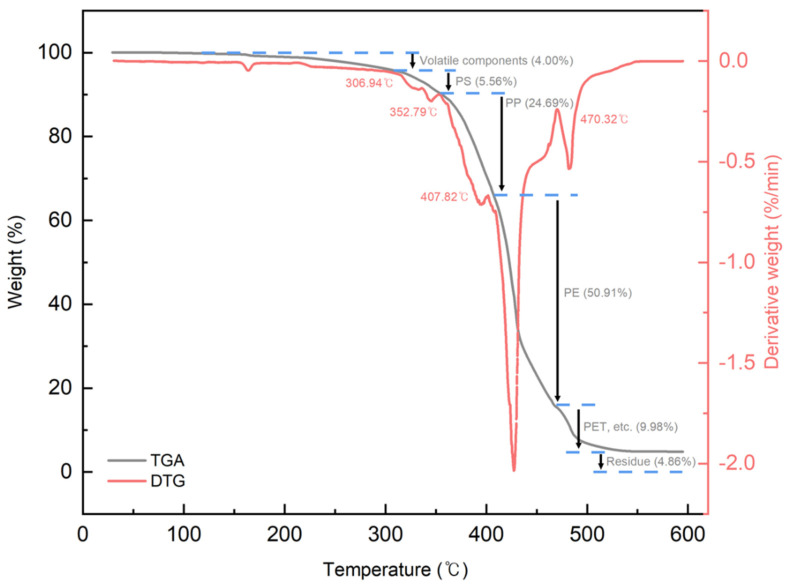
TGA and DTG measurement results of PCR plastics.

Figure 4 shows the FT-IR spectrum of the PCR plastic. In the FT-IR measurement results of the PCR plastic, specific peaks of PP and PE can be observed at the same positions, as shown in Figure 3. The contents of PP and PE were quantitatively analyzed by comparing the areas of the rPP, the rPE, and the PCR plastic, which revealed that the PCR plastic contained 28.95% PP and 71.05% PE, approximately in a ratio of 3:7. Figure 5 shows the results of the TGA and derivative thermogravimetric (DTG) measurements of the rPP and rPE. The DTG graph in (a) shows that the rPP started degradation at 351.06 °C, with maximum degradation occurring at 459.78 °C. The DTG graph in (b) shows that the rPE started degradation at 41.17 °C, with maximum degradation occurring at 475.41 °C. Both samples showed 1.94% and 2.30% of heterogeneous substances after degradation, respectively. Figure 6 shows the results of the TGA and DTG measurements of the PCR plastic. The degradation of PP and PE in the PCR plastic started at a temperature similar to the degradation temperature observed in Figure 5, according to the DTG results. The degradation of PP resulted in a weight loss of 24.69%, while the degradation of PE resulted in a weight loss of 50.91%. The FT-IR and DTG results confirmed the components within the PCR plastics in similar ratios. The thermal degradation temperature and content of the heterogeneous substances are crucial factors to consider due to the typical mixture of various materials in PCR plastics. The persistence of aluminum foil, even after complete thermal degradation, allows for residue measurement. Polyolefins were reported to undergo degradation within a temperature range of 300–500 °C in a study by Korol et al. [33]. Specifically, PP tends to degrade around 440–460 °C, while PE degrades around 460–475 °C [34,35,36,37]. Consistent with these findings, the observed thermal degradation of the PCR plastic confirms the presence of PP and PE. It has been reported that the residue content is usually less than 1 wt% when virgin polyolefins are completely degraded [38,39]. However, in the case of the PCR plastic, a residue content of 4.86% is observed. This higher residue content suggests that the properties of the PCR plastic may be lower compared to virgin plastics [40].

**Figure 7:**
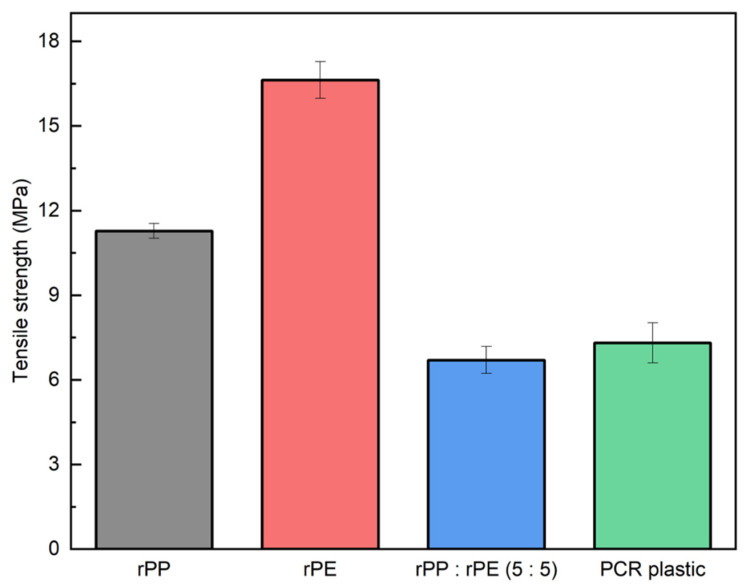
Tensile strength measurement results of rPP, rPE, rPP:rPE (5:5), and PCR plastic samples.

Figure 7 shows the tensile strengths of the rPP, rPE, rPP:rPE (5:5), and PCR plastic samples. The tensile strengths of the rPP and rPE were measured at 11.28 MPa and 16.63 MPa, respectively. In contrast, the tensile strength of the simply mixed rPP:rPE (5:5) sample was measured to be 6.70 MPa, while the PCR plastic exhibited a strength of 7.31 MPa. Figure 4 shows that the ratio of PP to PE in PCR plastics is approximately 3:7. However, the ratio of PP to PE is 5:5. In general, PCR plastics exhibit various ratios of PP and PE contents, such as 1:9, 2:8, and 3:7. In this experiment, we chose a representative 5:5 ratio to analyze. The recycling process for PCR plastic uses a mixture of different materials. Therefore, it is lower than the mathematically expected result obtained by simple mixing, as shown in Figure 7. Although PP and PE share similar hydrocarbon structures, they are thermodynamically immiscible, resulting in the formation of a binary system that compromises the strength [41,42]. Most PCR plastics consist not only of PP and PE but also a mixture of other plastics such as PET and PS. Instances of low strength due to immiscibility among different materials can be found in various other cases as well [43,44]. This makes it difficult to use the PCR plastics, so improving their strength is essential for expanding the applications of PCR plastics.

### 3.2. Optimization of Coupling Agents

**Figure 8:**
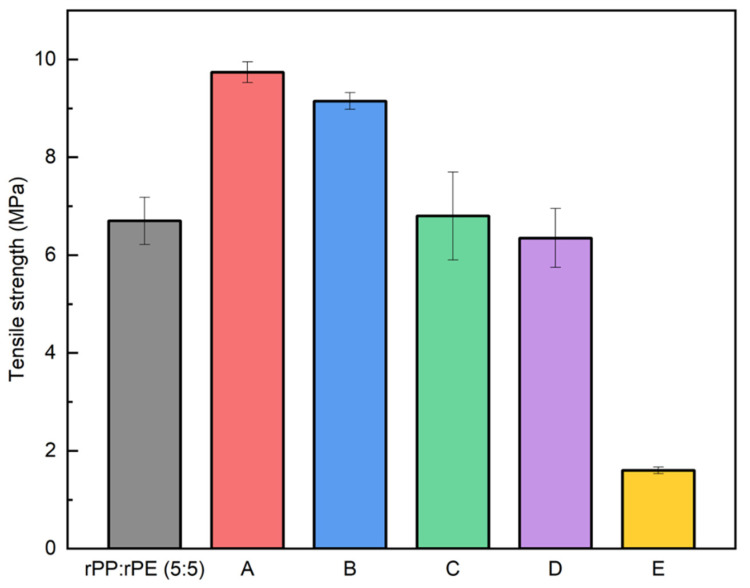
Tensile strength measurement results of rPP:rPE (5:5) sample with the application of commercial coupling agents.

As previously mentioned, PP and PE, despite sharing a similar hydrocarbon structure, do not thermodynamically mix and form a binary system. To enhance the material properties, a coupling agent was applied. The coupling agent provides strong interfacial interactions for property enhancement, offers the possibility of chemical bonding, and improves stress transfer and interfacial adhesion, thereby inhibiting material delamination and improving properties. Therefore, the application of a coupling agent can greatly enhance the performance of binary plastic systems [45,46,47]. Figure 8 shows the tensile strength results of the rPP:rPE (5:5) sample with 5 wt% of commercial coupling agents. Commercial coupling agents, labeled as A to E in Table 1, were used. Coupling agents A and B showed a significant increase in tensile strength, while no improvement was observed with C, D, and E. Particularly, coupling agent A exhibited an increase of approximately 45% with a tensile strength of 9.74 MPa. This was attributed to its higher grafting ratio (5 wt%), which improved the interfacial interaction between PP and PE compared to the other coupling agents.

**Figure 9:**
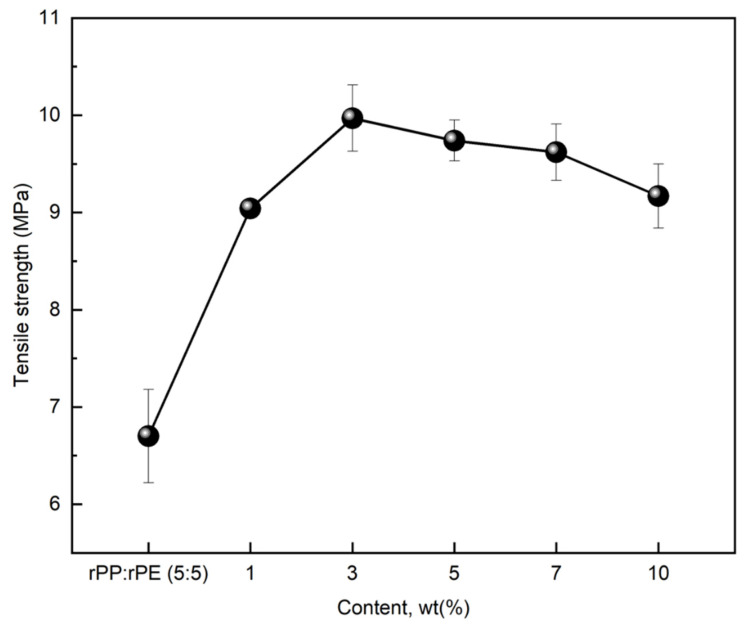
Changes in tensile strength of rPP:rPE (5:5) sample according to coupling agent A content.

The application of 5 wt% of coupling agent A to the rPP:rPE (5:5) sample resulted in the highest increase in tensile strength. However, coupling agents can be applied at various concentrations, allowing for the adjustment of properties, and therefore, it is necessary to find the optimized concentration for effective performance. Figure 9 shows the results of measuring the tensile strength of the rPP:rPE (5:5) sample by applying 1, 3, 5, 7, and 10 wt% of coupling agent A to find the optimal content. Coupling agent A exhibited an increase in tensile strength up to 3 wt%, after which it started to decrease from 5 wt% onwards. At 3 wt%, the tensile strength exhibited the highest enhancement, with an increase of approximately 49% to reach 9.97 MPa.

**Figure 10:**
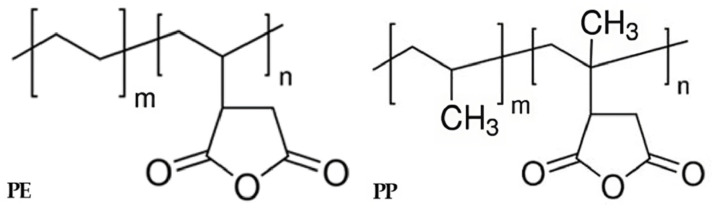
Coupling agent interaction schemes.

The chemical formula representing the interfacial interaction of the coupling agent, as shown in Figure 10, indicates the effective role of coupling agent application in improving tensile strength. However, the excessive use of a coupling agent can lead to the formation of agglomerates or clusters within the material, disrupting its overall homogeneity and uniformity and thereby reducing its mechanical properties. Additionally, it can hinder the mobility and movement of polymer chains within the material, limiting its ability to deform and absorb stress, resulting in decreased strength and toughness. Furthermore, an excessive concentration of the coupling agent can lead to the formation of excessive cross-links or chemical bonds within the material. While some degree of cross-linking can enhance the properties of the material, an excessive amount can create a rigid and brittle network, diminishing its flexibility and impact resistance [48,49]. Therefore, the application of coupling agents proves effective in enhancing the strength of immiscible materials, and finding the appropriate concentration ensures the prevention of property deterioration and obtaining the optimal strength.

**Figure 11:**
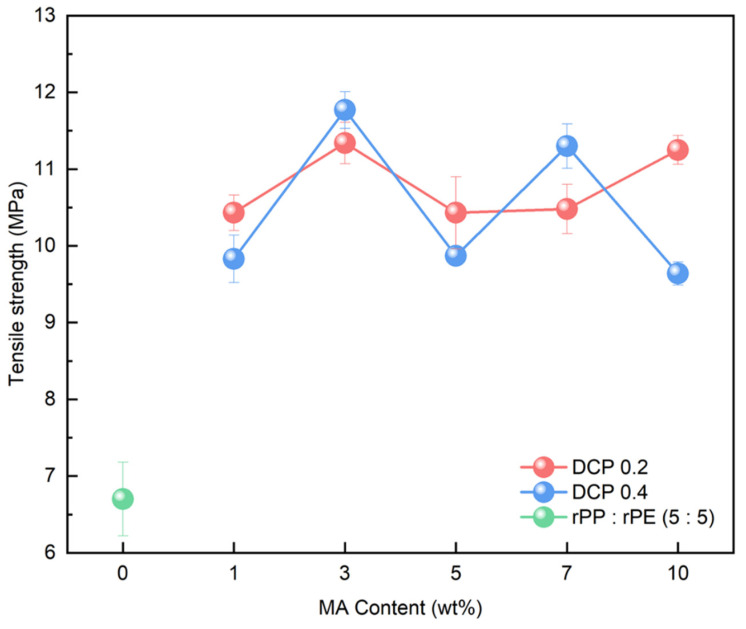
Tensile strength measurement results of rPP:rPE (5:5) sample with respect to the variation in the content of the MA coupling agent.

**Figure 12:**
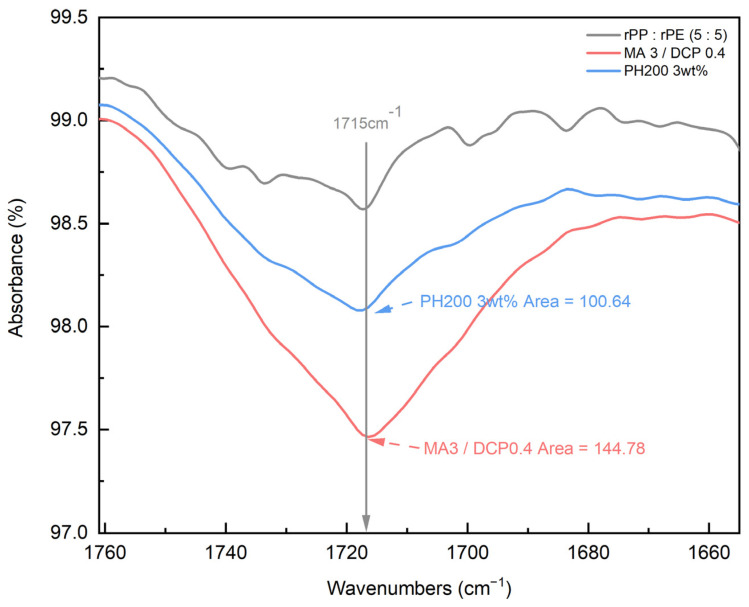
Grafting ratio measurement results (at 1715 cm^−1^) of coupling agent A and the MA coupling agent using FT-IR.

Coupling agent A exhibited the highest increase in tensile strength at 3 wt%. To compare the characteristics of coupling agent A with an MA coupling agent, we produced an MA coupling agent by adjusting the content of MA and DCP, requiring the identification of the optimal content, as shown in Table 2. The produced MA coupling agent was applied to the rPP:rPE (5:5) sample, and the tensile strength is depicted in Figure 11. The application of the MA coupling agent to the rPP:rPE (5:5) sample showed an observed improvement in tensile strength, surpassing coupling agent A for all content variations. Particularly, the highest enhancement was achieved with 3 wt% of MA and 0.4 wt% of DCP, which is the same content as coupling agent A. The difference in tensile strength between the MA coupling agent and coupling agent A can be determined by the grafting ratio measured through FT-IR. Figure 12 shows the grafting ratio difference in the rPP:rPE (5:5) sample for each coupling agent. The grafting ratio was determined by calculating the area under the peak observed at 1715 cm^−1^ [50]. The area for coupling agent A was measured to be 100.64, while for the MA coupling agent, it was measured to be 144.78. This discrepancy in area suggests a difference in grafting strength. Grafting strength ultimately determines the interfacial interaction strength, and a higher grafting ratio results in increased strength, ultimately leading to an improvement in tensile strength. As a result, the optimal content for the MA coupling agent was found to be 3 wt% of MAand 0.4 wt% of DCP, yielding the highest tensile strength and grafting ratio. Furthermore, unlike commercially available coupling agents that are typically produced with a fixed composition ratio, it can be concluded that the MA coupling agent exhibits optimized strength tailored to specific conditions that manufacturers may overlook by adjusting the appropriate ratio of MA and DCP and the synthesis conditions.

### 3.3. Application of Coupling Agents to PCR Plastics

**Figure 13:**
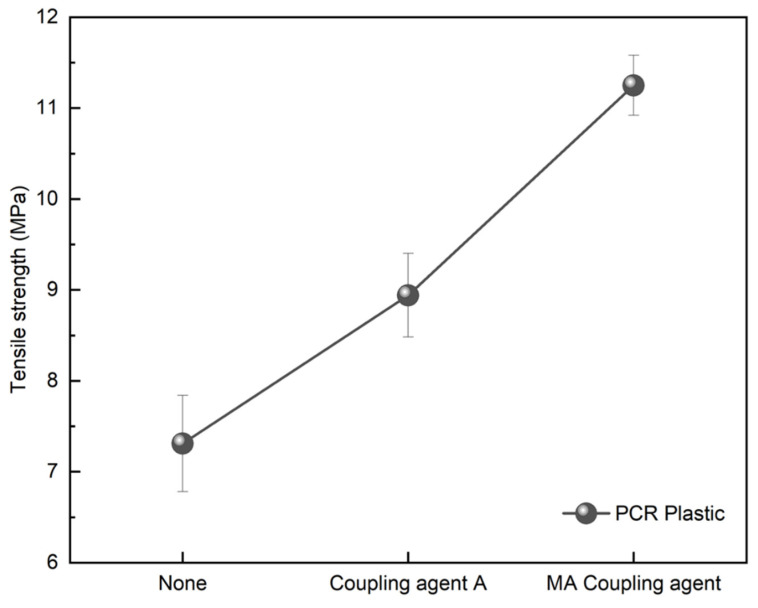
Tensile strength variation of PCR plastic with a coupling agent application.

Figure 9 and Figure 11 demonstrate the effective enhancement of tensile strength when 3 wt% of the coupling agent was applied to the immiscible rPP:rPE (5:5) blend. To improve the properties of the PCR plastic containing immiscible components, coupling agents were employed. Figure 13 shows the tensile strength of the PCR plastic treated with 3 wt% of coupling agents, including coupling agent A and the MA coupling agent. The tensile strength of the untreated PCR plastic was 7.31 MPa, while coupling agent A exhibited a 22.3% increase, reaching 8.94 MPa. However, the MA coupling agent showed a significant improvement of 53.9% with a tensile strength of 11.25 MPa. These results were consistent with those of the rPP:rPE (5:5) blend case, indicating a substantial increase in tensile strength attributed to the high grafting ratio of the MA coupling agent, as shown in Figure 12. The application of a coupling agent serves as a bridge between immiscible materials. Such coupling agent properties have been reported to enhance the fluidity of the material [51,52]. Table 4 shows the MFI of the materials with and without coupling agent application. The MFI values of rPP and rPE measured at 230 °C and 2.16 kg were 12.82 and 1.08 g/10 min, respectively. The rPP:rPE (5:5) blend showed an MFI of 4.51 g/10 min. With the application of coupling agent A and the MA coupling agent, the MFI increased by an average of 16.08% to 5.33 and 5.14 g/10 min, respectively. Additionally, the MFI of the PCR plastic was measured at 4.92 g/10 min, which significantly increased to 25.66 g/10 min, representing a maximum improvement of 421.5% upon the application of the MA coupling agent. In general, an increase in the MFI value indicates enhanced molecular mobility between polymer chains, which can be attributed to improved chain transfer or changes in the molecular weight distribution. Additionally, the application of coupling agents can lead to lubrication or plasticization effects [53]. Furthermore, applying the optimized MA coupling agent resulted in a significant increase in the MFI, indicating enhanced melt flow properties.

### 3.4. Application of Graphene to PCR Plastics

**Figure 14:**
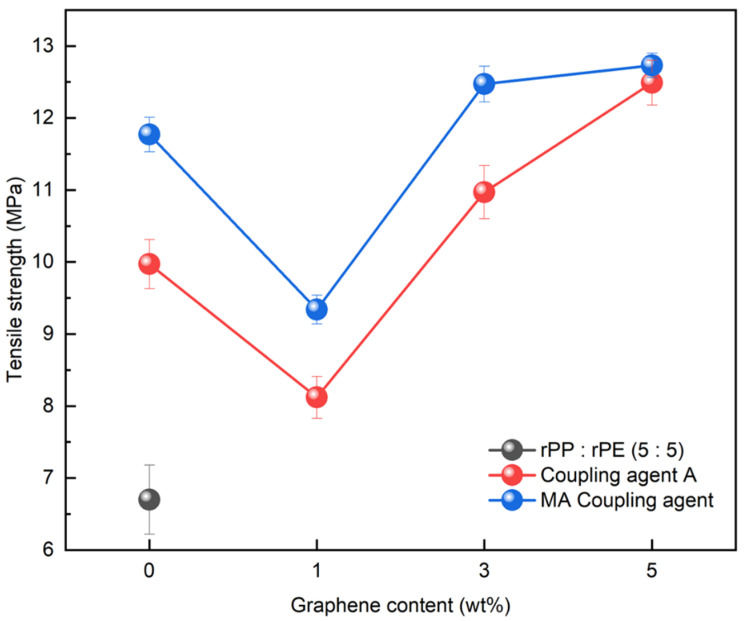
Changes in tensile strength of rPP:rPE (5:5) blend according to graphene content for coupling agent A and the MA coupling agent.

**Figure 15:**
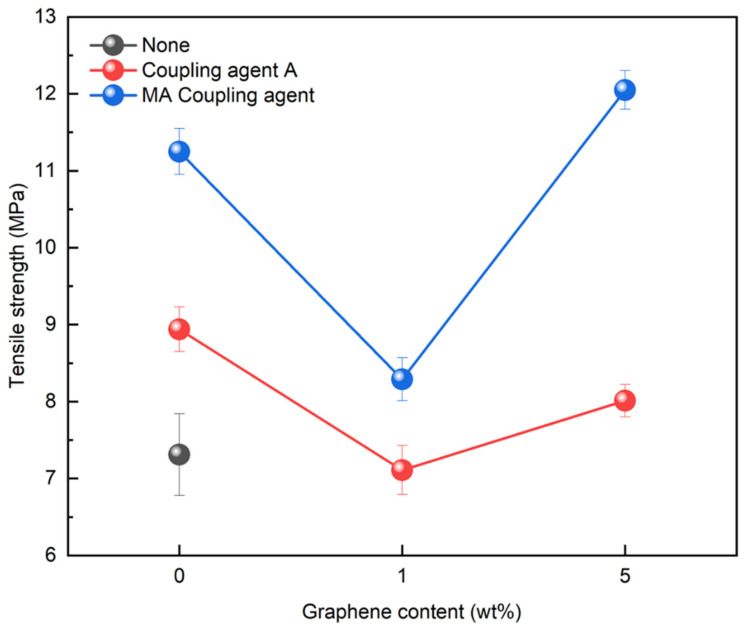
Changes in tensile strength of PCR plastics when graphene is used.

**Figure 16:**
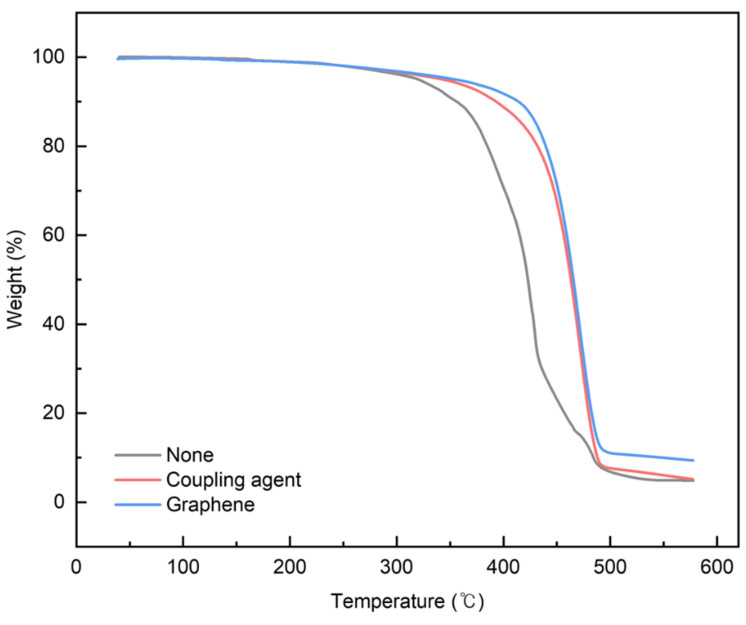
Weight changes in PCR plastics with coupling agent and graphene used.

Graphene, with its strong inherent bonding, can be utilized to improve mechanical properties in various applications. Therefore, its optimal concentration was investigated by applying it to the rPP:rPE (5:5) blend. Figure 14 shows the variation in the tensile strength with the application of the coupling agent and the presence or absence of graphene. At 1 wt%, the tensile strength initially decreased; however, it reached its highest value when the graphene content reached 5 wt%. It is known that an excessive use of nanofillers such as graphene can have a negative effect on strength [54]. Therefore, graphene was applied at up to 5 wt% for the evaluation. The increase in tensile strength when applying the MA coupling agent and graphene was higher compared to coupling agent A. The effect of the graphene concentration on the PCR plastic was examined by comparing the 1 wt% and 5 wt% graphene concentrations. Figure 15 shows the changes in the tensile strength with the application of the coupling agent and the presence or absence of 1 wt% and 5 wt% of graphene. When applying the MA coupling agent and 1 wt% of graphene, a decrease similar to that for the rPP:rPE (5:5) blend was observed. At lower graphene concentrations, the interaction between graphene particles may be limited; however, as the concentration increases, the particles interact more closely and form stronger bonds with the polymer [55]. Therefore, an increase in tensile strength was observed when applying 5 wt% of graphene. However, when using coupling agent A, no significant increase in the tensile strength was observed. This can be attributed to the higher grafting ratio of the MA coupling agent, maximizing the crosslinking effect and achieving stronger bonding. The application of the high-grafting-ratio MA coupling agent combined with 5 wt% of graphene resulted in a significant improvement in the tensile strength of the PCR plastic. Figure 16 shows the thermal degradation temperature change of the PCR plastic in the presence of the MA coupling agent and graphene. The use of MA coupling agents and graphene increased the thermal degradation temperature of the PCR plastics. This indicates that the application of MA coupling agents improves the compatibility of PP and PE, resulting in improved thermal stability [56,57]. In addition, the high thermal conductivity of graphene improves its thermal stability within the polymer matrix [58,59,60]. The improvement in tensile strength and thermal stability is expected to broaden the applications of PCR plastics.

## 4. Conclusions

PCR plastics composed primarily of PP and PE exhibit poor properties due to their immiscibility, making it challenging to enhance their performance. Therefore, this research aimed to improve the properties of PCR plastics by utilizing a coupling agent and graphene. The characterization through the FT-IR and TGA analyses revealed a PP:PE ratio of approximately 3:7 in the PCR plastic, with 4.86% heterogeneous materials present. A comparative analysis was conducted between commercially available coupling agents and a directly synthesized MA coupling agent. When applied to a 5:5 blend of rPP and rPE, the MA coupling agent outperformed its commercial counterparts, achieving the highest tensile strength at a concentration of 3 wt%. The addition of 3 wt% of both the commercial and MA coupling agents led to a significant 53.9% increase in the tensile strength, reaching 11.25 MPa, and a remarkable 421.54% increase in the MFI, reaching 25.66 g/10 min. Moreover, the incorporation of 5 wt% of graphene resulted in a notable 64.84% increase in the tensile strength. In addition, the use of the MA coupling agent and graphene improved the thermal stability of the PCR plastics. These findings hold significant promise for addressing environmental concerns associated with plastic waste by facilitating the recycling of PCR plastics into new products.

## Figures and Tables

**Table 1:** List of commercially available coupling agents used in this study.

No.	Trade Name	Abbreviation	MFI	Producer
A	PH-200	PP-g-MA (5% of MA)	100 g/10 min	Lotte Chemical, Seoul, Republic of Korea
B	EM-520	PP-g-LLDPE	2.3 g/10 min	Lotte Chemical, Seoul, Republic of Korea
C	CM-1120H	PP-g-MA (1% of MA)	70 g/10 min	Lotte Chemical, Seoul, Republic of Korea
D	NB2713A	PE-g-LLDPE	2–4 g/10 min	Woosung Chemical, Yeongcheon, Republic of Korea
E	SP2000S	Impact modifier (Nylon)	2–4 g/10 min	Woosung Chemical, Yeongcheon, Republic of Korea

**Table 2:** MA, DCP, and graphene mixing ratio.

Additive	Content (wt%)
MA	1, 3, 5, 7, 10
DCP	0.2, 0.4
Graphene	1, 3, 5

**Table 3:** rPP, rPE, and rPP:rPE (5:5) FT-IR spectral peak area.

Wavenumber, cm^−1^	Area	Area, rPP:rPE (5:5)
718 (PE)	344.92	216.74
1375 (PP)	439.00	290.52
2847 (PE)	1463.46	1004.22
2950 (PP)	1172.91	690.69

**Table 4:** Changes in MFI with the application of a coupling agent.

Type	Coupling Agent	MFI (g/10 min)
rPP	-	12.82
rPE	-	1.08
rPP:rPE (5:5)	-	4.51
	A	5.33
	MA	5.14
PCR plastic	-	4.92
	A	9.68
	MA	25.66

## Data Availability

Data are contained within the article.

## References

[1] Maxwell J. (1994). Plastics in the Automotive Industry.

[2] Lux J.H. (1994). Plastics in the space age. J. Franklin Inst..

[3] Lubin G., Dastin S.J. (1982). Aerospace Applications of Composites.

[4] Borrelle S.B., Ringma J., Law K.L., Monnahan C.C., Lebreton L., McGivern A., Rochman C.M. (2020). Predicted growth in plastic waste exceeds efforts to mitigate plastic pollution. Science.

[5] Adyel T.M. (2020). Accumulation of plastic waste during COVID-19. Science.

[6] Ncube L.K., Ude A.U., Ogunmuyiwa E.N., Zulkifli R., Beas I.N. (2021). An overview of plastic waste generation and management in food packaging industries. Recycling.

[7] Baran B. (2022). Resource (in) efficiency in the EU: A case of plastic waste. Ekon. Prawo.

[8] Su Y., Zhang Z., Wu D., Zhan L., Shi H., Xie B. (2019). Occurrence of microplastics in landfill systems and their fate with landfill age. Water Res..

[9] Verma R., Vinoda K.S., Papireddy M., Gowda A.N.S. (2016). Toxic pollutants from plastic waste-a review. Procedia Environ. Sci..

[10] He Q.G., Huang C.Y., Chang H., Nie L.B. (2013). Progress in recycling of plastic packaging wastes. Adv. Mat. Res..

[11] Tajeddin B., Arabkhedri M. (2020). Polymer and Food Packaging.

[12] Qureshi M.S., Oasmaa A., Pihkola H., Deviatkin I., Tenhunen A., Mannila J., Laine-Ylijoki J. (2020). Pyrolysis of plastic waste: Opportunities and challenges. J. Anal. Appl. Pyrolysis.

[13] Zhang G.H., Zhu J.F., Okuwaki A. (2007). Prospect and current status of recycling waste plastics and technology for converting them into oil in China. Resour. Conserv. Recycl..

[14] Macosko C.W. (2000). Morphology development and control in immiscible polymer blends. Macromol. Symp..

[15] Teh J.W., Rudin A., Keung J.C. (1994). A review of polyethylene–polypropylene blends and their compatibilization. Adv. Polym. Technol..

[16] Krigbaum W.R., Dawkins J.V., Via G.H., Balta Y.I. (1966). Effect of strain on the thermodynamic melting temperature of polymers. J. Polym. Sci. A-2: Polym. Phys..

[17] Drozdov A.D., Agarwal S., Gupta R.K. (2005). The effect of temperature on the viscoelastic response of polymer melts. Int. J. Eng. Sci..

[18] Kelly A.L., Gough T., Whiteside B.R., Coates P.D. (2009). High shear strain rate rheometry of polymer melts. J. Appl. Polym. Sci..

[19] Nachtigall S.M., Cerveira G.S., Rosa S.M. (2007). New polymeric-coupling agent for polypropylene/wood-flour composites. Polym. Test..

[20] Wah C.A., Choong L.Y., Neon G.S. (2000). Effects of titanate coupling agent on rheological behaviour, dispersion characteristics and mechanical properties of talc filled polypropylene. Eur. Polym. J..

[21] Ahmadlouydarab M., Chamkouri M., Chamkouri H. (2020). Compatibilization of immiscible polymer blends (R-PET/PP) by adding PP-g-MA as compatibilizer: Analysis of phase morphology and mechanical properties. Polym. Bull..

[22] Tucker J.D., Lee S., Einsporn R.L. (2000). A study of the effect of PP-g-MA and SEBS-g-MA on the mechanical and morphological properties of polypropylene/nylon 6 blends. Polym. Eng. Sci..

[23] Kiran M.D., Govindaraju H.K., Jayaraju T., Kumar N. (2018). Effect of fillers on mechanical properties of polymer matrix composites. Mater. Today Proc..

[24] Kar G.P., Biswas S., Bose S. (2015). Tailoring the interface of an immiscible polymer blend by a mutually miscible homopolymer grafted onto graphene oxide: Outstanding mechanical properties. Phys. Chem. Chem. Phys..

[25] Cao Y., Zhang J., Feng J., Wu P. (2011). Compatibilization of immiscible polymer blends using graphene oxide sheets. ACS Nano.

[26] (2023). Standard Test Method for Melt flow Rates of Thermoplastics by Extrusion Plastometer.

[27] Caban R. (2022). FTIR-ATR spectroscopic, thermal and microstructural studies on polypropylene-glass fiber composites. J. Mol. Struct..

[28] Na C.K., Park G.Y., Park H. (2018). Polypropylene surface with antibacterial property by photografting 1-vinylimidazole and subsequent chemical modification. Korean J. Chem. Eng..

[29] Takafuji M., Ide S., Ihara H., Xu Z. (2004). Preparation of poly (1-vinylimidazole)-grafted magnetic nanoparticles and their application for removal of metal ions. Chem. Mater..

[30] El-Hamshary H., Fouda M.M., Moydeen M., El-Newehy M.H., Al-Deyab S.S., Abdel-Megeed A. (2015). Synthesis and antibacterial of carboxymethyl starch-grafted poly (vinyl imidazole) against some plant pathogens. Int. J. Bio. Macromol..

[31] Dulal N., Shanks R., Gengenbach T., Gill H., Chalmers D., Adhikari B., Martinez I.P. (2017). Slip-additive migration, surface morphology, and performance on injection moulded high-density polyethylene closures. J. Colloid Interface. Sci..

[32] Stuart B.H. (2004). Infrared Spectroscopy: Fundamentals and Applications.

[33] Korol J., Hejna A., Wypiór K., Mijalski K., Chmielnicka E. (2021). Wastes from agricultural silage film recycling line as a potential polymer materials. Polymers.

[34] Contat-Rodrigo L., Ribes-Greus A., Imrie C.T. (2002). Thermal analysis of high-density polyethylene and low-density polyethylene with enhanced biodegradability. J. Appl. Polym. Sci..

[35] Das P., Tiwari P. (2018). Valorization of packaging plastic waste by slow pyrolysis. Resour. Conserv. Recycl..

[36] Rego A., Silva A.S., Grillo A.V., Santos B.F. (2019). Thermogravimetric Study of Raw and Recycled Polyethylene Using Genetic Algorithm for Kinetic Parameters Estimation. Chem. Eng. Trans..

[37] Shafigullin L.N., Romanova N.V., Gumerov I.F., Gabrakhmanov A.T., Sarimov D.R. (2018). Thermal properties of polypropylene and polyethylene blends (PP/LDPE). IOP Conf. Ser. Mater. Sci. Eng..

[38] Sharma S.K., Nema A.K., Nayak S.K. (2010). Polypropylene nanocomposite film: A critical evaluation on the effect of nanoclay on the mechanical, thermal, and morphological behavior. J. Appl. Polym. Sci..

[39] Aboulkas A., El Bouadili A. (2010). Thermal degradation behaviors of polyethylene and polypropylene. Part I: Pyrolysis kinetics and mechanisms. Energy Convers. Manag..

[40] Thoden van Velzen E.U., Chu S., Alvarado Chacon F., Brouwer M.T., Molenveld K. (2021). The impact of impurities on the mechanical properties of recycled polyethylene. Packag. Technol. Sci..

[41] Graziano A., Titton Dias O.A., Sena Maia B., Li J. (2021). Enhancing the mechanical, morphological, and rheological behavior of polyethylene/polypropylene blends with maleic anhydride-grafted polyethylene. Polym. Eng. Sci..

[42] Graziano A., Jaffer S., Sain M. (2019). Review on modification strategies of polyethylene/polypropylene immiscible thermoplastic polymer blends for enhancing their mechanical behavior. J. Elastomers. Plast..

[43] Jose S., Thomas S., Parameswaranpillai J., Aprem A.S., Karger-Kocsis J. (2015). Dynamic mechanical properties of immiscible polymer systems with and without compatibilizer. Polym. Test..

[44] Jiang G., Wu H., Guo S. (2010). Reinforcement of adhesion and development of morphology at polymer–polymer interface via reactive compatibilization: A review. Polym. Eng. Sci..

[45] Koning C., Van Duin M., Pagnoulle C., Jerome R. (1998). Strategies for compatibilization of polymer blends. Prog. Polym. Sci..

[46] Pracella M., Haque M.M.U., Alvarez V. (2010). Functionalization, compatibilization and properties of polyolefin composites with natural fibers. Polymers.

[47] Clarke R.W., Sandmeier T., Franklin K.A., Reich D., Zhang X., Vengallur N., Chen E.Y.X. (2023). Dynamic crosslinking compatibilizes immiscible mixed plastics. Nature.

[48] Durmaz B.U., Aytac A. (2020). Characterization of carbon fiber-reinforced poly (phenylene sulfide) composites prepared with various compatibilizers. J. Compos. Mater..

[49] Li H., Xie X.M. (2017). Morphology development and superior mechanical properties of PP/PA6/SEBS ternary blends compatibilized by using a highly efficient multi-phase compatibilizer. Polymer.

[50] Sclavons M., Carlier V., De Roover B., Franquinet P., Devaux J., Legras R. (1996). The anhydride content of some commercial PP-g-MA: FTIR and titration. J. Appl. Polym. Sci..

[51] Kong Y., Li Y., Hu G., Cao N., Ling Y., Pan D., Shao Q., Guo Z. (2018). Effects of polystyrene-b-poly (ethylene/propylene)-b-polystyrene compatibilizer on the recycled polypropylene and recycled high-impact polystyrene blends. Polym. Adv. Technol..

[52] Abacha N., Fellahi S. (2005). Synthesis of polypropylene-graft-maleic anhydride compatibilizer and evaluation of nylon 6/polypropylene blend properties. Polym. Int..

[53] Ahmed K., Raza N.Z., Habib F., Aijaz M., Afridi M.H. (2013). An investigation on the influence of filler loading and compatibilizer on the properties of polypropylene/marble sludge composites. J. Ind. Eng. Chem..

[54] Lodhi R.S., Kumar P., Achuthanunni A., Rahaman M., Das P. (2022). Mechanical properties of polymer/graphene composites. Polymer Nanocomposites Containing Graphene.

[55] Jiang L.Y., Huang Y., Jiang H., Ravichandran G., Gao H., Hwang K.C., Liu B. (2006). A cohesive law for carbon nanotube/polymer interfaces based on the van der Waals force. J. Mech. Phys. Solids.

[56] Jana R.N., Mukunda P.G., Nando G.B. (2003). Thermogravimetric analysis of compatibilized blends of low density polyethylene and poly (dimethyl siloxane) rubber. Polym. Degrad. Stab..

[57] Akhlaghi S., Sharif A., Kalaee M., Elahi A., Pirzadeh M., Mazinani S., Afshari M. (2012). Effect of stabilizer on the mechanical, morphological and thermal properties of compatibilized high density polyethylene/ethylene vinyl acetate copolymer/organoclay nanocomposites. Mater. Des..

[58] Fim F.D.C., Basso N.R., Graebin A.P., Azambuja D.S., Galland G.B. (2013). Thermal, electrical, and mechanical properties of polyethylene–graphene nanocomposites obtained by in situ polymerization. J. Appl. Polym. Sci..

[59] Mittal V., Chaudhry A.U. (2015). Polymer-graphene nanocomposites: Effect of polymer matrix and filler amount on properties. Macromol. Mater. Eng..

[60] Wan Y.J., Yang W.H., Yu S.H., Sun R., Wong C.P., Liao W.H. (2016). Covalent polymer functionalization of graphene for improved dielectric properties and thermal stability of epoxy composites. Compos. Sci. Technol..

